# Dynamic network link prediction with node representation learning from graph convolutional networks

**DOI:** 10.1038/s41598-023-50977-6

**Published:** 2024-01-04

**Authors:** Peng Mei, Yu hong Zhao

**Affiliations:** School of Information Engineering, Inner Mongolia University of Science and Technology, Baotou, 014010 China

**Keywords:** Mathematics and computing, Computer science

## Abstract

Dynamic network link prediction is extensively applicable in various scenarios, and it has progressively emerged as a focal point in data mining research. The comprehensive and accurate extraction of node information, as well as a deeper understanding of the temporal evolution pattern, are particularly crucial in the investigation of link prediction in dynamic networks. To address this issue, this paper introduces a node representation learning framework based on Graph Convolutional Networks (GCN), referred to as GCN_MA. This framework effectively combines GCN, Recurrent Neural Networks (RNN), and multi-head attention to achieve comprehensive and accurate representations of node embedding vectors. It aggregates network structural features and node features through GCN and incorporates an RNN with multi-head attention mechanisms to capture the temporal evolution patterns of dynamic networks from both global and local perspectives. Additionally, a node representation algorithm based on the node aggregation effect (NRNAE) is proposed, which synthesizes information including node aggregation and temporal evolution to comprehensively represent the structural characteristics of the network. The effectiveness of the proposed method for link prediction is validated through experiments conducted on six distinct datasets. The experimental outcomes demonstrate that the proposed approach yields satisfactory results in comparison to state-of-the-art baseline methods.

## Introduction

The objective of link prediction for dynamic networks is to evaluate the probability of future connections between nodes. Owing to the rapid advancement of communication networks, the Internet, and the big data era, dynamic network analysis has emerged as a crucial research problem, attracting the attention of experts from various fields towards dynamic network link prediction. In biology, dynamic network link prediction has many potential applications in protein network modeling, including: Protein interaction prediction^1^, evolution of metabolic network^2^, and conversion mechanism of signal transduction between proteins provide conditions for guiding the control of diseases^3^ and signal transduction complexes^4^, as well as revealing the interaction relationship between molecules, which provides potential value for drug design^5^, disease understanding and gene regulation^6^. These applications demonstrate the important role of dynamic network link prediction in protein network modeling, which helps to understand the structure and function of protein interaction networks from a dynamic perspective, and provides new insights and methods for biological research. In the social domain^7^, we focus on the network evolution of social users, which is of great significance for social media marketing, information dissemination^8^ research, and social dynamic analysis. Moreover, dynamic network link prediction is not limited to social media and can be applied to various fields such as finance, transportation networks, and environmental science. It aids in better understanding crucial changes, event evolution, and node behavior within the network, thereby offering support for diverse application scenarios.

The existing works on dynamic network link prediction face two primary challenges: First, employing a single neighbor information to represent nodes overlooks the influence of node clustering, neighbor relationship, and time evolution in the network. Second, when constructing temporal attribute models, the spotlight is often narrowed down to the evolution pattern of the global time step, neglecting the impact of short-term connections and feature changes between nodes and their neighbors in a single time step, which can affect the accuracy of node embedding vectors. To address the aforementioned issues, this study presents a GCN_MA framework. In this paper, a NRNAE algorithm is proposed to enrich the node information representation by using node degree, clustering coefficient and neighbor relationship, so that GCN can aggregate the above multi-dimensional features to learn node embedding vectors more comprehensively. RNN and multi-head attention are used to model the time attributes from the global and local perspectives respectively, so as to complete the link prediction task more accurately and comprehensively. In terms of global time attribute modeling, the whole time step is considered, and the parameters $W$ of GCN are continuously updated through Long Short-Term Memory (LSTM) network, which can dynamically adjust the aggregation of multi-dimensional features of GCN in each time step to learn the global time evolution information. Local temporal attribute modeling focuses on a specific time step, captures the changes of links and features around the node and its neighbors through multi-head attention, and makes adaptive adjustments to the node embedding vector, so as to obtain high-quality node embedding vector representation. The contributions of this paper are as follows:

1. This study presents a GCN-based node representation learning framework that captures temporal attributes by examining global and local information fluctuations, thereby offering a more comprehensive depiction of the temporal evolution pattern of dynamic networks.
2. A novel NRNAE algorithm is proposed to enrich the structural features of the network and ensure the effectiveness of the aggregated information learned by GCN at each node.
3. Comprehensive experiments were conducted to validate the efficacy of the GCN_MA framework in dynamic network link prediction and to compare it to several advanced baseline methods.

## Related research

In recent years, a multitude of dynamic network link prediction techniques have been proposed by researchers. Among these, the method grounded in similarity measurement is deemed to be the most straightforward and potent. This approach posits that the higher the similarity between nodes, the greater the likelihood of their forming connections^9^. In recent years, a multitude of dynamic network link prediction techniques have been proposed by researchers. Among these, the method grounded in similarity assessment is deemed as the most straightforward and potent. In this approach, the higher the degree of similarity between nodes, the greater the likelihood of establishing links^9^. Chen et al.^10^ introduced NCC and NCCP, two similarity measures founded on the clustering coefficient of neighboring nodes. Wu et al.^11^ advanced a technique capable of dynamically predicting the similarity of future node pairs, and calculated the similarity measure for nodes via an algorithm based on node ranking. Zhang et al.^12^ proposed a method integrating node centrality with time series to appraise the impact of common neighbors in dynamic networks, and to capture the evolving pattern of node centrality over time. The proficiency of graph convolutional networks in learning intricate systems has prompted some researchers to develop representation learning techniques based on this approach. Cui et al.^13^ introduced DyGCN, a variant of GCN that caters to dynamic networks by updating node embeddings to propagate embedding information. Chami et al.^14^ merged GCN with hyperbolic geometry, designing hyperbolic graph convolutional neural networks (HGCN) to acquire inductive node representations for hierarchical and scale-free graphs.

With the ongoing advancements in deep learning technology research, an increasing number of studies have emerged that focus on applying deep learning techniques for dynamic network link prediction. Xian et al.^15^ proposed a link prediction model, GraphLP, based on network reconstruction theory, which leverages the feature learning capability of deep learning models to automatically extract structural patterns from graphs for improved link prediction. In an effort to capture the evolution patterns of time, Zhou et al.^16^ employed the concept of triadic closure as a guiding principle to capture the evolution patterns of various snapshots. Goyal et al.^17^ introduced DynGEM, a model based on deep autoencoder, which progressively updates node embeddings from the initialization of the previous step. However, these methods struggle with capturing long-term dynamics, resulting in limitations to their accuracy. To address this issue, Chen et al.^18^ proposed a novel end-to-end model, GC-LSTM, which effectively combines LSTM and GCN to capture the local structural attributes of nodes and their interrelationships for enhanced link prediction in dynamic networks. The above-mentioned methods primarily rely on global time information for modeling temporal attributes but do not take into account the more intricate changes in local information of nodes within dynamic networks. Therefore, this study needs to consider modeling temporal attributes from the perspective of local information alterations to comprehensively capture temporal evolution patterns.

## Definitions and methods

### Definition of a dynamic network

A dynamic network can be represented as a sequence of discrete snapshots, denoted as ${\text{G}} = \{ {\text{G}}_{1} ,G_{2} , \ldots ,G_{T} \}$, where $G_{t} = (V,E_{t} ,A_{t} )$ $(t \in [1,T])$ represents the t-th time network snapshot. Let $V$ be the set of all nodes and $E_{t}$ denote the set of edges within a fixed time interval $[t - \tau ,t]$. $A_{t}$ denotes the adjacency matrix of $G_{t}$, where $A_{t} (i,j) = 1$ if there is a link between nodes $i$ and $j$, and $A_{t} (i,j) = 0$ otherwise.

### Definition of link prediction in dynamic networks

The link prediction in dynamic networks^19^ aims to forecast the adjacency matrix $\widehat{A}_{T + 1}$ corresponding to the next time step snapshot $G_{T + 1}$ at time step $T + 1$, given the prior $T$ historical network snapshots $G = \{ G_{1} ,G_{2} , \ldots ,G_{T} \}$ along with their respective adjacency matrices $\{ A_{1} ,A_{2} , \ldots ,A_{T} \}$, as illustrated in Eq. (1):

1 $$\widehat{A}_{T + 1} = f(A_{1} ,A_{2} , \ldots ,A_{T} )$$

**Figure 1:**
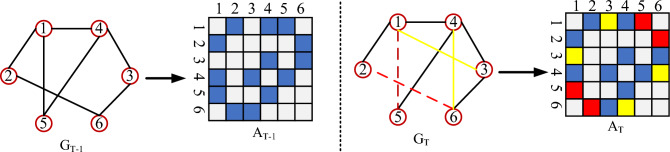
Dynamic network evolution and an illustration of its adjacency matrix. The network time evolves from time $T - 1$ to time $T$ when E (2,6) and E (1,5) disappear and E (1,3) and E (4,6) appear and are filled with red and yellow colors in the corresponding adjacency matrix.

The model to be constructed is represented by $f( \cdot )$, while $\widehat{A}_{T + 1} \in R^{N \times N}$ denotes the predicted value. The topology of a dynamic network evolves over time, and its progression can be exemplified by the alterations in the adjacency matrix across different time intervals, as illustrated in Fig. 1. The objective of dynamic network link prediction is to identify the links that are most likely to emerge or vanish in the subsequent time window. In the $A_{T}$ context, yellow denotes the newly established connection, while red signifies the disintegrated link.

### Node representation learning framework based on graph convolutional network

**Figure 2:**
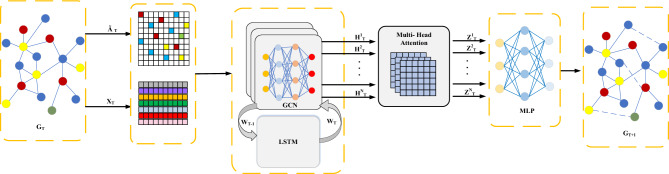
Illustration of the overall framework of dynamic network link prediction based on GCN_MA. The NRNAE algorithm is used to represent the node information, GCN is used to aggregate multi-dimensional features to learn the embedding vector representation of the node, and LSTM and Multi-Head Attention are used to capture the global and local time evolution patterns respectively. Finally, MLP is used to calculate the probability value of the edge to complete the link prediction task.

In this study, a node representation learning architecture grounded on GCN, coined GCN_MA, is introduced. The design of this architecture seeks to integrate the multi-dimensional features of node degree, clustering coefficient, and time evolution to comprehensively and accurately represent node information, while simultaneously modeling the time attribute from both global and local perspectives. This enables a more comprehensive learning of the time evolution pattern of dynamic networks, ultimately achieving the objective of link prediction. In the following, the main components of the proposed framework are detailed, including node information representation, learning of node representation vectors, and temporal property modeling, as illustrated in Fig. 2.

Firstly, the NRNAE algorithm was initially employed to mine network information, thereby enriching the network structure features $\{ \widetilde{A}_{1} ,\widetilde{A}_{2} , \ldots ,\widetilde{A}_{T} \}$. The node degree matrix was utilized as the node features $\{ X_{1} ,X_{2} , \ldots ,X_{T} \}$ to describe node information from two perspectives. The learning of node representation vectors involves aggregating multi-dimensional features via GCN to acquire each node embedding vector representation $\{ H_{T}^{1} ,H_{T}^{2} , \ldots ,H_{T}^{N} \}$, where N signifies the number of nodes. In this study, an improved LSTM was incorporated to continuously update the parameter *W* of GCN. This enables GCN to dynamically adjust the aggregation of multi-dimensional features across different time steps, effectively modeling the time attribute of dynamic graphs from a global perspective. To further explore the dynamic characteristics of dynamic networks, a multi-head attention mechanism was introduced. This mechanism adaptively assigns reasonable weights to each node embedding vector, enabling the learning of local structure information for each time snapshot from a local perspective. Ultimately, it generates a new node embedding vector representation $Z_{T} = \{ Z_{T}^{1} ,Z_{T}^{2} , \ldots Z_{T}^{N} \}$. The synergy of these two mechanisms allows the framework to capture the nuances of time evolution with higher accuracy. To complete the link prediction task for dynamic networks, the probability value of chain edges between nodes is calculated based on a Multi-Layer Perceptron (MLP).

### Node information representation

The evolution of dynamic networks over time involves assessing not only the impact of individual nodes on the network's overall structure, but also the influence on neighboring nodes. This includes the tendency of neighboring nodes to cluster and the level of interaction between neighbors and other nodes. Consequently, this study defines the concept of "node aggregation effect," which quantifies the influence of the clustering tendency between a node's attributes and its neighbors on the connection strength between the node and its neighboring nodes at different time steps. The node clustering effect encompasses multidimensional features such as degree and clustering coefficient. To harness the network information for node representation and enable GCN to learn more accurate node embedding vectors by aggregating multi-dimensional features, in this paper, a Node Representation Algorithm Based on Node Aggregation Effect (NRNAE) is proposed.

$CC(i)$ (clustering coefficient, CC) stands for clustering coefficient^20^, which is used to describe the degree of clustering between a vertex $i$ and other nodes in a graph; specifically, it captures the degree to which the neighbors of a target node are interconnected, within the range of $CC(i) \in [0,1][0,1]$, as illustrated in Eq. (2):

2 $$CC(i) = \frac{{2R_{i} }}{{K_{i} (K_{i} - 1)}}$$

where $R_{i}$ denotes the number of triangles formed by node $i$ and its first-order neighbor nodes, and $K_{i}$ denotes the number of first-order neighbor nodes of $i$. In complex networks, the degree of a node quantifies the node's significance, which can be comprehended as the cumulative influence of edges surrounding the node. Additionally, the clustering coefficient is employed to gauge the extent of clustering among nodes in the network. In an endeavor to scrutinize the traits of nodes more exhaustively, this study defines a novel concept, referred to as " Aggregation Strength ".

Aggregation Strength (AS)$AS(i)$ is used to describe the probability of focusing on a node to form a cluster, and reflects the importance and influence of the node. The specific definition is illustrated in Eq. (3):

3 $$AS(i) = deiree(i) * CC(i)$$

where $\deg ree(i)$ denotes the degree of node $i$ and $CC(i)$ denotes the clustering coefficient of node $i$.

The Node Aggregation Effect is utilized to describe the extent to which the attributes of a node and the propensity of neighboring nodes to cluster influence the connection strength between the node and its neighbors throughout the dynamic evolution process. The aggregation effect of nodes $i$ and $j$ is determined based on the Aggregation Strength. The specific definition is illustrated in Eq. (4).

4 $$S(i,j) = |{\rm N}(i) \cap {\rm N}(j)| * AS(i)$$

where, ${\rm N}(i)$ denotes the set of first-order neighbor nodes of node $i$, and $j \in {\rm N}(i)$. ${\rm N}(j)$ represents the set of first-order neighbor nodes of node $j$. $S(i,j)$ is normalized by $S(i,j) \in [0,1]$. $S_{T} \in R^{N \times N}$ represents the matrix of node aggregation effects ($t \in [1,T]$) constructed by the $S_{T} (i,j)$. Furthermore, to examine the contribution of $S_{T}$ to the aggregated feature information of GCN, a weighting factor $\beta \in [0,1]$ is incorporated. In this study, a node representation algorithm based on node aggregation effect is employed to create a new adjacency matrix $\widetilde{\rm A}_{T}$, which comprehensively represents node information. This enables GCN to aggregate multi-dimensional feature information and enhance the accuracy of the learned node embedding vector, as illustrated in Eq. (5):

5 $$\widetilde{\rm A}_{T} = {\rm A}_{T} + \beta S_{T}$$

where, ${\rm A}_{T} \in R^{N \times N}$ represents the adjacency matrix of snapshots at time step $T$.

### Nodes represent the learning of vectors

Graph convolutional networks have powerful learning capabilities for complex systems and can effectively deal with graph data structures with non-Euclidean features. The core idea of Graph Convolutional Networks (GCN)^21^ is to extend deep learning methods to graph-structured data in order to effectively learn and represent the relationships between nodes while preserving the topological structure of nodes on the graph. Compared with the graph convolutional network based on spatial domain, the graph convolutional network based on spectral domain is more suitable for dealing with various complex graph data, and can consider the global structure information of the whole graph. In this study, we utilize the graph convolutional network based on the spectral domain to calculate the embedding vector representation $H_{T} = \{ H_{T}^{1} ,H_{T}^{2} , \ldots ,H_{T}^{N} \}$ for each node at a specific time step *T*, as illustrated in Eq. (6).

6 $$H_{T} = GCN(A_{T} ,S_{T} ,X_{T} |W_{T} ) = \sigma \left( {\widehat{D}_{T}^{{ - \frac{1}{2}}} \widehat{S}_{T} \widehat{D}_{T}^{{ - \frac{1}{2}}} X_{T} W_{T} } \right)$$

where,${\rm A}_{T} \in R^{N \times N}$ and $X_{T} \in R^{N \times f}$ represent the adjacency matrix and node characteristic matrix of $G_{T}$, respectively, and $\widehat{D}_{T} \in R^{N \times N}$ is the diagonal matrix generated by $\widehat{D}_{i,j} = \sum\nolimits_{j} {\widehat{S}_{i,j} }$. $\widehat{S}_{T} = {\rm A}_{T} + \beta S_{T} + I_{N}$, with $I_{N} \in R^{N \times N}$ as the identity matrix. $\sigma$ symbolizes the ReLU function that introduces nonlinearity, and $W_{T} \in R^{f \times D}$ is the weight parameter that can be learned. This paper proposes a new aggregation strategy for ${\rm A}_{T} + \beta S_{T} + I_{N}$ that is different from the traditional GCN method that uses ${\rm A}_{T} + I_{N}$ to guide node feature aggregation. In this paper, the node aggregation effect $S_{T}$ is added to further guide GCN to aggregate network structure information. Under the new aggregation strategy, the target node can identify the degree of influence of different neighbor nodes on its importance, and use this importance to guide GCN aggregation.

### Global time attribute modeling based on LSTM

In this study, we establish a node representation matrix $H_{T}$ for a specific time step. Due to the independent learning of $H_{T}$ for each specific time step $T$, these matrices are mutually exclusive. These low-dimensional representation matrices solely capture the local network structure information at a specific time step, lacking the ability to encapsulate global structural information and dynamic network time evolution patterns. To address these issues, we require an effective strategy to simulate the temporal properties across different time steps, enabling the node representation matrix $H_{T}$ to learn the global structural information of the graph from all time steps and resulting in high-quality node embedding vector representations.

**Figure 3:**
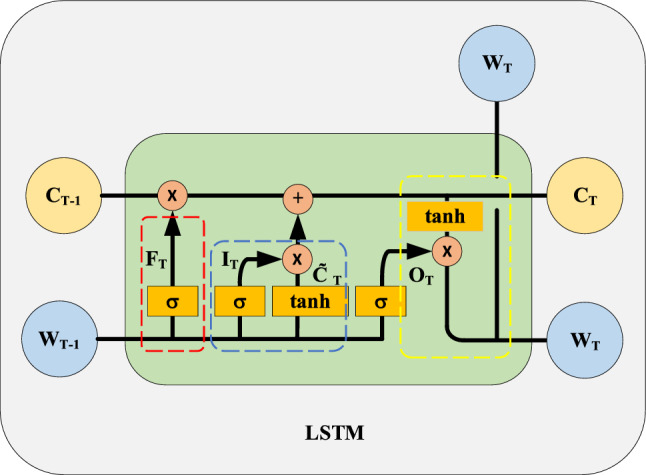
Update process of parameters in GCN by LSTM. The LSTM updates the weight parameter $W_{T}$ of the GCN at time step $T$ based on the previous $T - 1$ time steps.

The LSTM^22^ architecture exhibits flexible nonlinear transformation capabilities while processing time series data, enabling it to uncover the temporal evolution patterns of dynamic networks. In an effort to enhance the model's temporal expression capabilities, an improved LSTM will be employed to capture the time attribute of dynamic graphs. In this paper, all time steps are considered for the global time attribute modeling, and the parameters of GCN are continuously updated through LSTM, and the learning of these parameters is also passed to GCN after timely training and learning in LSTM, so that the aggregation of multi-dimensional features of GCN can be dynamically adjusted in each time step to learn the global time evolution information. As shown in Fig. 3, in this study, the weight matrix $W_{T - 1}$ generated by the GCN at the previous time step is utilized as the input to the LSTM to produce the weight matrix $W_{T}$ at the subsequent time step, as illustrated in Eq. (7):

7 $$W_{T} = LSTM(W_{T - 1} )$$

Given $\widetilde{\rm A}_{T} \in R^{N \times N}$, $X_{T} \in R^{N \times f}$, and $W_{T - 1} \in R^{f \times D}$, the LSTM is extended to the matrix version by constructing a node embedding vector matrix $H_{T}$ according to Eqs. (3) and (7). The update process, realized through Eqs. (8)–(13), is elucidated as follows:

8 $$F_{T} = \sigma (M_{F} W_{T - 1} + U_{F} W_{T - 1} + b_{F} )$$

9 $$I_{T} = \sigma (M_{I} W_{T - 1} + U_{I} W_{T - 1} + b_{I} )$$

10 $$O_{T} = \sigma (M_{O} W_{T - 1} + U_{O} W_{T - 1} + b_{O} )$$

11 $$\widetilde{C}_{T} = \tanh (M_{C} W_{T - 1} + U_{C} W_{T - 1} + b_{C} )$$

12 $$C_{T} = F_{T} \circ C_{T - 1} + I_{T} \circ \widetilde{C}_{T}$$

13 $$W_{T} = O_{T} \circ \tanh (C_{T} )$$

where,$U_{\psi } \in R^{f \times f}$ and $M_{\psi } \in R^{f \times f}$ are weight matrices.$b_{\psi } \in R^{f \times D}$ is a bias vector, $\psi \in \{ F,I,O,C\}$. As shown in Fig. 3, $F_{T} \in R^{f \times D}$ represents the computation of the forget gate in the LSTM, which determines how much information needs to be retained in the cell state at the previous time step. $I_{T} \in R^{f \times D}$ is the computation of the input gate that determines which new temporal information will be added to the cell state. $O_{T} \in R^{f \times D}$ is the computation of the output gate, which determines which time information can be output. $\widetilde{C}_{T} \in R^{f \times D}$ is the updating process of the cell state. In the context of this paper's framework, the GCN weight parameter *W* are solely updated by the enhanced LSTM, negating the need for further training and learning. Consequently, the number of parameters in the GCN does not escalate with the time step, thereby reducing both time and space complexity. Moreover, the framework continuously updates the GCN weight parameter *W* through the LSTM in each time step. This dynamic adjustment of the weight parameter *W* enables the GCN to better adapt to the time evolution and enhances the learning of node embedding vectors in graph data. Subsequent ablation experiments also confirmed that updating the GCN parameters through LSTM indeed improves the accuracy of link prediction.

### Local time attribute modeling based on multi-head attention

In the field of dynamic network link prediction research, the alterations in local information within dynamic graphs are often overlooked, such as the rapid shifts in link status and node characteristics of a node and its neighboring nodes. Nevertheless, patterns that capture the evolution of local information can provide a more refined depiction of the dynamics within dynamic networks. The Multi-Head attention^21^ model assumes a crucial role in this context, as it can effectively capture the interrelationships between nodes and neighbors, making it particularly suitable for modeling local information. By utilizing the multi-head attention model, it becomes feasible to focus on distinct neighbor nodes in each head, thereby facilitating a more comprehensive understanding of the local environment of the node and further assisting in the capture of the temporal evolution pattern of the dynamic network.

In this study, the node embedding vector $H_{T}$ at time step *T* is employed as the input for the multi-head attention model in this study, where $H_{T} = \{ H_{T}^{1} ,H_{T}^{2} , \ldots ,H_{T}^{N} \}$. The scaled dot product attention, as adopted from Ref^23^, is utilized to accelerate computation. For each time step, the temporal attention layer accepts $H_{T}$ as input and generates a novel sequence of node embedding vectors, denoted as $Z_{T} = \{ Z_{T}^{1} ,Z_{T}^{2} , \ldots Z_{T}^{N} \}$. In this research, the multi-head attention model is utilized to learn features from diverse latent spaces, thereby enhancing the model's representation capacity^24^. Within each attention head, a linear transformation is applied to the input vector to produce $Q_{T}$, $K_{T}$, and $V_{T}$, as illustrated in Eqs. (14–16):

14 $$Q_{T} = W_{q} H_{T} \in R^{{D_{K} \times N}}$$

15 $$K_{T} = W_{k} H_{T} \in R^{{D_{K} \times N}}$$

16 $$V_{T} = W_{v} H_{T} \in R^{{D_{V} \times N}}$$

The attention weights are computed using $Q_{T}$ and $K_{T}$ and are subsequently applied to $V_{T}$ to obtain the output $Attention(Q_{T} ,K_{T} ,V_{T} )$, as illustrated in Eq. (17):

17 $$Attention(Q_{T} ,K_{T} ,V_{T} ) = soft\max (\frac{{Q_{T} K_{T}^{T} }}{{\sqrt {d_{k} } }})V_{T}$$

where $Q_{T}$, $K_{T}$, and $V_{T}$ are three matrices, and $Q_{T} \in R^{{D_{K} \times N}}$, $K_{T} \in R^{{D_{K} \times N}}$, $V_{T} \in R^{{D_{V} \times N}}$.$\sqrt {d_{K} }$ are scaled.

**Figure 4:**
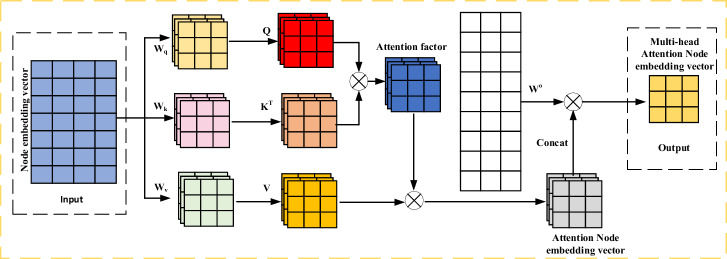
Data flow of head attention model. The input is the node embedding vector $H_{T}$ at time $T$, and the input is mapped to $Q_{T} ,K_{T} ,V_{T}$ by linear variation. For each head, the attention weight coefficient is calculated, and it is applied to the corresponding $V_{T}$ to obtain the attention output $head_{i}$ of each head. Then, the output of multiple heads is concatenated, and the new node embedding vector $Z_{T}$ is finally output.

Figure 4 demonstrates the implementation of the multi-head attention mechanism, which involves processing the original input sequence through multiple sets of self-attention operations. Subsequently, the results of each self-attention set are concatenated and undergo a linear transformation to produce the final output $Z_{T} = \{ Z_{T}^{1} ,Z_{T}^{2} , \ldots Z_{T}^{N} \}$. The specific computation is delineated in Eqs. (18–19):

18 $$head_{i} = Attention(QW_{i}^{Q} ,KW_{i}^{K} ,VW_{{\text{i}}}^{V} )$$

19 $$Z_{T} = Concat(head_{1} ,head_{2} , \ldots ,head_{h} )W^{o}$$

where $Z_{T} \in R^{N \times D}$, $W_{i}^{Q} \in R^{{D_{K} \times N}}$, $W_{i}^{K} \in R^{{D_{K} \times N}}$, $W_{i}^{V} \in R^{{D_{V} \times N}}$ and $W^{o} \in R^{{hD_{V} \times N}}$ are parameter matrices.

### Link prediction and loss function

The link prediction problem is reformulated as a binary classification task in this study, with the Multilayer Perceptron (MLP)^25^ serving as the classifier. The embedding vector $Z_{T} = \{ Z_{T}^{1} ,Z_{T}^{2} , \ldots Z_{T}^{N} \}$. of nodes, which is learned through the effective integration of GCN, LSTM, and multi-head attention models, is employed as the input to the MLP. By learning the nonlinear relationship between data through the hidden layer, a probability matrix $P_{T}$ is ultimately generated via the output layer, as illustrated in Eq. (20).

20 $$P_{T} = \sigma (MLP(Z_{T} ))$$

$MLP( \cdot )$ represents a fully connected network endowed with parameters, which include an input layer, a hidden layer, and an output layer. $Z_{T} \in R^{N \times D}$ serves as the input to $MLP( \cdot )$, while *ReLU* acts as the activation function in the hidden layer and $\sigma$ as the activation function for the softmax () in the output layer. $P_{T} \in R^{N \times N}$ constitutes a probability matrix built from $P_{T} (i,j)$. A higher value of $P_{T} (i,j)$ corresponds to a greater likelihood of an edge existing between nodes *i* and *j*. Conversely, $P_{T} (i,j) = 0$ signifies the absence of edges between nodes.

The binary cross-entropy loss function^26^, which evaluates the accuracy of model predictions and facilitates online parameter updates without the need for retraining the entire model, as illustrated in Eq. (21):

21 $$Loss = - \frac{1}{{N^{2} }}\sum\limits_{i = 1}^{{N^{2} }} {(Y_{T} \log (P_{T} )) + (1 - Y_{T} )\log (1 - P_{T} )}$$

where *N* is the total number of samples, $P_{T}$ is the probability matrix, and $Y_{T} \in \{ 0,1\}$ represents the labels of the target links indicating whether the links exist.

## Results and analysis

### Dataset

To assess the efficacy of the proposed framework, experiments are performed on six real-world datasets. The comprehensive statistics of the six datasets are delineated in Table 1.

**Table 1:** Statistics of the dataset.

Data type	Number of nodes	Number of edges	Time steps
CollegeMsg	1899	59,835	47
Mooc_actions	7047	411,749	72
Bitcoinotc	6005	35,592	62
EUT	1005	332,334	127
LastFM	1000	1,293,103	76
Wikipedia	5684	87,931	42

CollegeMsg^27^: It represents message communication among users of the online community of students at the University of California, Irvine. Mooc_action^28^: The MOOC user actions dataset represents actions performed by users on popular MOOC platforms. Bitcoinotc^29^: The Who Trusts Who network of people who transact with Bitcoin on a platform called Bitcoin OTC. Email-eu-core-temporal (EUT)^30^: This network is the institution generated using Email data from a large European study. LastFM^31^: This dataset is based on the concept of the original Last.fm dataset and is based on the million-song dataset. Wikipedia^32^: This public dataset is edited for a month by editors on Wikipedia pages. We have selected a variety of datasets from distinct domains, such as social interactions, communication patterns, financial transactions, and Wikipedia, among others. These datasets encompass both sparse and dense networks, which aids in evaluating the effectiveness of the proposed method more thoroughly, given the diversity of the data.

### Baseline methods

DGCN^33^ introduced a novel dice similarity to overcome the problem of unclear directional neighbor influence, further guiding aggregation, and updating GCN's weight parameters with Long Short-Term Memory (LSTM) to capture global structural information for all time steps in dynamic graphs.

HTGN^34^ maps temporal graphs into hyperbolic space and combines hyperbolic graph neural networks and hyperbolic gated recurrent neural networks to capture evolving behaviors while implicitly preserving hierarchical information. It successfully models temporal networks by employing the Hyperbolic Temporal Self-Attention (HTA) module to focus on historical states and the Hyperbolic Temporal Consistency (HTC) module to ensure stability and generalization.

DyGNN^35^ models dynamic information as the evolution of a graph. Specifically, the proposed framework continuously updates node information by consistently capturing the sequence information of edges (interactions), as well as the time intervals between edges and information propagation.

EvolveGCN^36^ adjusts the GCN model along the time dimension without requiring assistance from node embeddings. The proposed method captures the dynamics of graph sequences by evolving GCN parameters using an RNN. In our comparison, we employ the improved Long Short-Term Memory (LSTM) network within RNN.

### Evaluation index

The accuracy of the algorithm was measured by the link prediction metrics AUC (Area Under Curve) and AP (Average Precision). They have different emphases on measuring prediction accuracy: AUC measures the overall accuracy of an algorithm; AP only considers whether the prediction is accurate for the top-L ranked edges. AUC can be interpreted as comparing a randomly selected edge from the test set with a randomly selected edge that is not present, and adding 1 point if the score value of the edge in the test set is greater than the score value of the edge that is not present. If the scores are equal, add 0.5 points. For $n$ independent comparisons, if there are $n^{\prime}$ times when the score value of the edge in the test set is greater than the score of the edge that is not present, and $n^{\prime\prime}$ times when the score value is equal, then the AUC is defined in Eq. (22):

22 $$AUC = \frac{{n^{\prime} + 0.5n^{\prime\prime}}}{n}$$

Precision is defined as the proportion of accurate predictions among the top *L* predicted edges. If *m* predictions are accurate, that is, *m* of the top *L* edges are in the test set, then Precision is defined in Eq. (23):

23 $$Pecision = \frac{m}{L}$$

### Analysis of experimental results

In this paper, we evaluate the results of the GCN_MA framework across different metrics and datasets. In addition, this paper investigates the impact of changing the parameter β in the NRNAE algorithm on the AUC and AP of the framework in link prediction, and validates the effectiveness of the proposed NRNAE algorithm for helping graph convolutional networks to aggregate node embedding vector representations. At the same time, this paper uses ablation experiments to verify the effectiveness of each component for the framework.

The experimental results presented in Table 2 demonstrate that GCN_MA yields optimal AUC and AP values across all six networks when compared to other baseline methods. Under the first four datasets, the AUC of GCN_MA surpasses 90%, reaching a remarkable 98.880% in the Mooc-action dataset. Although DGCN exhibits superior performance across six datasets, it falls short of the proposed method due to its inability to account for the influence of connectivity and time evolution between neighbors on node importance in dynamic networks. HTGN employs two hyperbolic spaces to capture historical state and time consistency. However, it overlooks the local and global characteristics of node features in dynamic networks, resulting in an AUC and AP of only 71.51% and 70.88% on the LastFM dataset. DyGCN utilizes the continuous time property to capture edge order information and update node information based on the time interval between information propagation. However, it only considers local time evolution, failing to capture global time evolution like LSTM, resulting in subpar performance on the ETU, Wikipedia, and LastFM datasets. The AUC of GCN_MA on other datasets improves by 5.38%, 2.93%, and 3.51% respectively. EvolveGCN enhances the GCN model by combining GRU and LSTM RNNs to learn time feature information of dynamic networks, yet it does not integrate node feature information with time feature information for node embedding vector representation. Consequently, the AUC and AP on the Wikipedia dataset stand at 62.89% and 61.63%, respectively. In comparison, the proposed framework in this paper achieves a notable improvement of 24.53% and 24.12% in AUC and AP, respectively.

**Table 2:** Comparison of link prediction AUC and AP of each method in different datasets.

Metric	Methods	Mooc-action	CollegeMsg	ETU	Bitcoinotc	LastFM	Wikipedia
AUC	DyGNN	0.9242	0.8856	0.7527	0.8769	0.8034	0.8371
	EvolveGCN	0.7794	0.7867	0.8494	0.7811	0.8593	0.6289
	HTGN	0.9712	0.8491	0.8694	0.8814	0.7151	0.8414
	DGCN	0.9720	0.8799	0.8947	0.9046	0.8201	0.8472
	GCN_MA	**0.9880**	**0.9149**	**0.9222**	**0.9120**	**0.8757**	**0.8742**
AP	DyGNN	0.9179	0.8839	0.7519	0.8617	0.8067	0.8371
	EvolveGCN	0.7602	0.7620	0.8453	0.7838	0.8566	0.6163
	HTGN	0.9773	0.8813	0.8642	0.8752	0.7088	0.8624
	DGCN	0.9657	0.8813	0.8788	0.8884	0.7781	0.8379
	GCN_MA	**0.9863**	**0.8926**	**0.9082**	**0.8943**	**0.8704**	**0.8575**

Best performing values are in bold.

The results show that combining the long short-term memory network with multi-head attention mechanism to capture the time evolution pattern of the dynamic network can indeed obtain the structural information of the network more detailed and more comprehensive, so as to better promote the link prediction of the dynamic network.

### Parametric analysis

**Figure 5:**
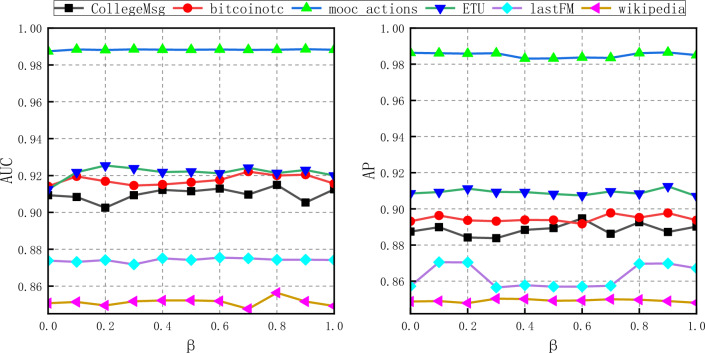
Curves of AUC and AP as a function of parameter *β* on six real data sets. The results show that when the value of *β* is between 0.7 and 0.9, the AUC and AP indicators of GCN_MA basically reach the optimal value at the same time.

To validate whether the proposed NRNAE algorithm can enhance the comprehensive fusion of node information by Graph Convolutional Networks (GCN), and thereby effectively improve the accuracy and precision of link prediction in dynamic networks, a series of experiments are conducted. In these experiments, 80% of the dataset is employed as the training set, while the parameter *β* in the algorithm is incrementally adjusted from 0.0 to 1.0 to assess its impact on the results. As illustrated in Fig. 5, the AUC and AP values of the Mooc-action dataset exhibit a significantly superior performance compared to those of the other datasets. This exceptional performance can be attributed to the higher dataset density, simpler data structure, and inclusion of more time segments, which facilitate a more comprehensive utilization of network information and thereby enhance the accuracy of the learned node embedding vectors. Consequently, the results demonstrate that the AUC and AP values are less sensitive to the parameter *β*.

In summary, when the value is between 0.7 and 0.9, the AUC and AP indices of GCN_MA concurrently achieve their optimal values, exhibiting an average increase of 0.52% and 0.45% in AUC and AP respectively, compared to the parameter value of 0, across six datasets. This finding suggests that the NRNAE algorithm, constructed considering the aggregation effect and aggregation strength, can enhance the information representation of nodes. Consequently, it aids GCN in integrating node embedding vectors more effectively, thereby enhancing the accuracy and precision of dynamic network link prediction.

### Ablation experiment

In this section, we conduct ablation experiments to analyze the contributions of each component in the GCN_MA model and the role played by each element in the model. Our study introduces three deep learning architectures in GCN_MA: graph convolutional network, long short-term memory network, and multi-head attention. To assess the impact of each component on the performance of GCN_MA, we sequentially remove one component and compare the results with the complete GCN_MA. The three ablation models are as follows: (1) GCN: This variant excludes the long short-term memory network and multi-head attention. (2) GCN_LSTM: This variant omits multi-head attention. (3) GCN_MultiAttention: This variant excludes the long short-term memory network.

**Figure 6:**
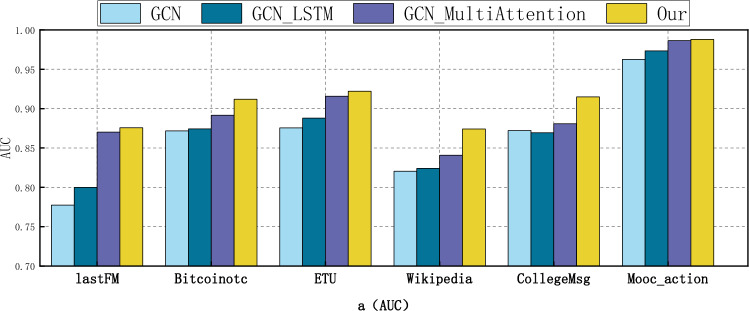
Comparison of GCN_MA and different ablation models in AUC on six real datasets.

**Figure 7:**
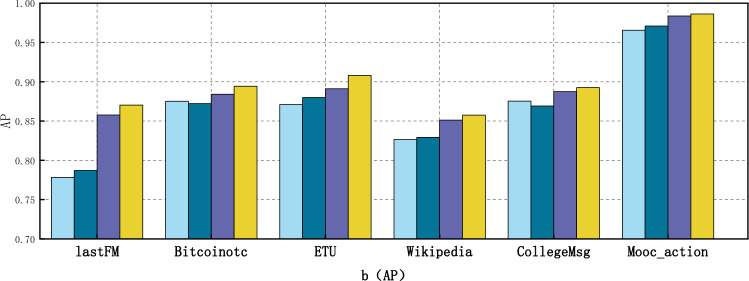
Comparison of GCN_MA and different ablation models in AP on six real datasets.

The dynamic network ablation experiments on six real datasets are presented in Tables 3, 4, and Figs. 6, 7. The figure demonstrates that all three components significantly contribute to the overall performance of GCN_MA. Specifically, GCN_MA exhibits an average improvement of 1.69% and 0.9% in terms of AUC and AP indicators, respectively, compared with GCN_MultiAttention. This suggests that the long short-term memory network is instrumental in capturing the global time evolution pattern. Furthermore, GCN_MA outperforms GCN_LSTM by an average of 4.3% and 3.34% in AUC and AP metrics, respectively. This indicates that the multi-head attention model plays a crucial role in capturing the temporal evolution pattern of local information changes in dynamic networks. Regarding AUC and AP indicators, GCN_MA averages a 5.12% and 3.62% increase compared to GCN. Thus, it can be inferred that the combination of long short-term memory network and multi-head attention is effective in modeling the time properties of dynamic networks, allowing for a more comprehensive capture of the time evolution pattern. When compared to GCN, GCN_LSTM, and GCN_MultiAttention, the average growth rates of AUC and AP for GCN_MA on six datasets are 6.11%, 5.08%, 1.93%, and 4.36%, 4.00%, 1.02%, respectively. Notably, GCN demonstrates the highest growth rate, indicating that it performs admirably in aggregating node information and significantly contributes to the link prediction of dynamic networks.

**Table 3:** AUC results of GCN_MA ablation experiments.

Methods	Dataset					
	lastFM	Bitcoinotc	ETU	Wikipedia	CollegeMsg	Mooc_action
**Ours**	**0.8757**	**0.9120**	**0.9222**	**0.8742**	**0.9149**	**0.9880**
GCN_MultiAttention	0.8702	0.8916	0.9158	0.8407	0.8809	0.9865
GCN_LSTM	0.7999	0.8743	0.8880	0.8241	0.8694	0.9734
GCN	0.7774	0.8718	0.8755	0.8205	0.8722	0.9626

Best performing values are in bold.

**Table 4:** AP results of GCN_MA ablation experiments.

Methods	Dataset					
	lastFM	Bitcoinotc	ETU	Wikipedia	CollegeMsg	Mooc_action
**Ours**	**0.8704**	**0.8943**	**0.9082**	**0.8575**	**0.8926**	**0.9863**
GCN_MultiAttention	0.8577	0.8842	0.8911	0.8511	0.8876	0.9838
GCN_LSTM	0.7872	0.8722	0.8780	0.8293	0.8692	0.9709
GCN	0.7782	0.8752	0.8711	0.8265	0.8754	0.9655

Best performing values are in bold.

The results in Table 3 indicate that the contribution of LSTM is indeed inferior to that of multi-head attention when compared with GCN_MultiAttention. This discrepancy arises because LSTM solely focuses on capturing the temporal evolution pattern within node embeddings. In contrast, multi-head attention not only captures the time evolution pattern but also adjusts the structural characteristics of the network by dynamically weighting neighbor nodes' influence on the target node based on their changes over time. This adjustment plays a crucial role in enhancing the accuracy of final node embedding vectors, thereby rendering multi-head attention more impactful than LSTM alone. However, combining both mechanisms further enhances link prediction accuracy.

In conclusion, GCN_MA, a model that synchronously integrates multi-dimensional features and analyzes time attributes from both global and local perspectives, demonstrates appreciable growth rates in AUC and AP indicators. This further validates the effectiveness of the proposed method in dynamic network link prediction.

## Conclusion

In this paper, we propose a node representation learning framework based on graph convolutional networks, called GCN_MA. The proposed framework aims to capture node information comprehensively and accurately, and model the temporal properties of dynamic networks from a global and local perspective to learn the temporal evolution patterns of the network and achieve link prediction. In this paper, NRNAE algorithm is used to enrich the node information representation, and GCN is used to aggregate structural features and node features, so as to learn the embedding vector representation of each node. At the same time, this paper introduces a recurrent neural network with multi-head attention to model the dynamic network from the perspective of global and local information changes, respectively, to capture the evolution pattern of time. We experimentally compare our framework with four baseline methods on six different datasets. The focus of this paper is on studying discrete dynamic networks with a homogeneous network type. However, in future work, we will shift our attention towards heterogeneous dynamic networks and time continuous dynamic networks in order to consider both structural similarity and feature-based similarity as measures for node similarity. This will enable us to obtain a higher quality low-dimensional representation that can effectively address the problem of dynamic network link prediction.

## Data Availability

The dataset used in this study is available at http://snap.stanford.edu/data/act-mooc.html, http://snap.stanford.edu/data/soc-sign-bitcoin-otc.html, http://snap.stanford.edu/data/email-Eu-core-temporal.html, https://meta.wikimedia.org/wiki/Data_dumps.
